# Widespread CNS pathology in amyotrophic lateral sclerosis homozygous for the D90A *SOD1* mutation

**DOI:** 10.1007/s00401-022-02519-z

**Published:** 2022-11-16

**Authors:** Karin M. Forsberg, Karin S. Graffmo, Erica Stenvall, Naima Tabikh, Stefan L. Marklund, Thomas Brännström, Peter M. Andersen

**Affiliations:** 1 Department of Clinical Sciences, Neurosciences, Umeå University, 90185 Umeå, Sweden; 2 Department of Medical Biosciences, Pathology, Umeå University, Umeå, Sweden; 3 Department of Medical Biosciences, Clinical Chemistry, Umeå University, Umeå, Sweden

**Keywords:** Amyotrophic lateral sclerosis, Superoxide dismutase-1, D90A, Neuronal inclusions, Human autopsy

## Abstract

Mutations in the gene encoding the ubiquitously expressed free radical scavenging enzyme superoxide dismutase-1 (*SOD1*) are found in 2–6% of amyotrophic lateral sclerosis patients. The most frequent *SOD1* mutation worldwide is D90A. Amyotrophic lateral sclerosis caused by this mutation has some unusual features: the heredity is usually recessive, the phenotype is stereotypic with slowly evolving motor symptoms beginning in the legs and may also include sensory, autonomic, and urinary bladder involvement. Furthermore, the mutant protein resembles the wild type, with normal content and enzymatic activity in the central nervous system. Here, we report neuropathological findings in nine patients homozygous for the D90A mutation. All nine had numerous small granular inclusions immunoreactive for misfolded SOD1 in motor neurons and glial nuclei in the spinal cord and brainstem. In addition to degeneration of the corticospinal tracts, all patients had degeneration of the dorsal columns. We also found intense gliosis in circumscribed cortical areas of the frontal and temporal lobes and in the insula. In these areas and in adjacent white matter, there were SOD1 staining neuropil threads. A few SOD1-immunopositive cytoplasmic neuronal inclusions were observed in cortical areas, as were glial nuclear inclusions. As suggested by the symptoms and signs and earlier neurophysiological and imaging investigations, the histopathology in patients homozygous for the D90A *SOD1* extends beyond the motor system to include cognitive and sensory cortical areas. However, even in the patients that had a symptomatic disease duration of more than 2 or 3 decades and lived into their 70s or 80s, there were no SOD1-inclusion pathology and no typical dysfunction (apart from the musculature) in non-nervous organs. Thus, only specific parts of the CNS seem to be vulnerable to toxicity provoked by homozygously expressed mutant SOD1.

**Supplementary Information:**

The online version contains supplementary material available at 10.1007/s00401-022-02519-z.

## Introduction

Amyotrophic lateral sclerosis (ALS) is a neurodegenerative disorder characterized by adult-onset degeneration of primarily upper and lower motor neurons resulting in progressive paresis that begins focally and spreads to adjacent myotomes [2, 39, 40]. Up to 13% of patients self-report a familial predisposition for ALS (denoted fALS), but genealogical studies reveal that at least 20–26% of patients have a familial predisposition for ALS or an ALS-like disease [1]. Mutations in the gene encoding the ubiquitously and constitutively expressed antioxidant enzyme superoxide dismutase-1 (*SOD1*) account for 12–23% of fALS and 2–6% of all ALS, with large differences across populations [1, 6, 42]. Since 1993, more than 220 mutations have been discovered in *SOD1*. The most prevalent is the D90A mutation (*SOD1*^*D90A*^*,* aka Asp90Ala, p.D91A, c.272A > C), which has been reported in 27 countries from Mongolia across Siberia, Afghanistan, Iran, and many European countries to Portugal [1, 3, 4]. In Western populations, *SOD1*^*D90A*^ is the second-most frequently identified cause of ALS (following *C9orf72HRE*). In most populations, ALS caused by *SOD1*^*D90A*^ is inherited as a recessive trait, and heterozygous members in these families do not develop ALS. All other ALS-associated *SOD1* mutations are inherited as a Mendelian dominant trait. In D90A *SOD1*-homozygous individuals (*SOD1*^*D90Ahom*^), the disease penetrance is high, with ages of onset ranging from 20 to 94 years (median of 46 years) [3, 4].

The hallmark of the phenotype in *SOD1*^*D90Ahom*^ individuals is a slowly progressing motor neuron disease beginning asymmetrically invariably in the lower limbs followed by slowly ascending paresis. The first observed motor sign is dysfunction in the upper motor neuron system (UMN), but later, affection of the lower motor neuron system (LMN) prevails, and severe wasting with a gradient from the feet to bulbar innervated muscles is the norm. A prodromal phase with sensory symptoms in the form of diffuse neuralgic lower back pain and arthralgia in the lower limbs and focal paraesthesia frequently precede the onset of progressive paresis by months or even years in some patients. Urgency of micturition and intermittent heat sensations have been observed in one-quarter of *SOD1*^*D90Ahom*^ patients. Some may also show intermittent cerebellar ataxia [1, 3]. Emotional lability may develop late during the disease course, but fulminant frontotemporal dementia (FTD) has not been reported in *SOD1*^*D90Ahom*^ patients. The phenotype thus deviates from that most commonly seen in ALS and resembles Patrikios’ disease (aka *Pseudopolyneuritic* variant of ALS) [11]. Corroborating that the phenotype is caused by the patient being homozygous for D90A *SOD1*, transgenic mice overexpressing D90A mutant human SOD1 develop a slowly evolving motor neuron disorder beginning in the hind legs and also involving bladder disturbance with incontinence [25].

In contrast to *SOD1*^*D90Ahom*^ individuals, a number of rare ALS patients heterozygous for *SOD1*^*D90A*^ have been reported [4]. These *SOD1*^*D90Ahet*^ patients usually report no familial predisposition for ALS and have never been observed in families where *SOD1*^*D90Ahom*^ individuals are affected with ALS. Interestingly, while some *SOD1*^*D90Ahet*^ patients present with a phenotype as seen in *SOD1*^*D90Ahom*^ patients, most *SOD1*^*D90Ahet*^ patients show a variable phenotype for site of onset of paresis (bulbar/arm/leg) and have a more aggressive disease course than in *SOD1*^*D90Ahom*^ patients [4, 30, 41, 44]. ALS patients’ compound heterozygous for D90A/another *SOD1* mutation has also been reported [14, 22]. Interestingly, they had slowly evolving phenotypes. The finding in several populations that heterozygous patients can be associated with a more malignant phenotype than homozygous patients is enigmatic.

In the present study, we prospectively identified, monitored, and eventually obtained autopsy tissues from nine *SOD1*^*D90*Ahom^ ALS patients. We used the material to investigate the pathomorphological pattern of SOD1^D90A^ inclusions.

## Materials and methods

### Patients

Tissues were collected from nine ALS patients homozygous for the D90A *SOD1* mutation [4] at the Department of Neurology, Umeå University Hospital. All patients fulfilled the El Escorial criteria for ALS. While EMG showed the typical neurogenic changes for ALS, transcranial magnetic stimulation of the motor cortex (MEP) revealed prolonged to very prolong central conductance latency in all patients, sometimes in asymptomatic limbs [3]. Clinical management was in accordance with the EFNS guidelines [2], and most patients eventually received a gastro-feeding tube when dysphagia and malnutrition became an issue and non-invasive ventilation through a face mask [9, 12]. None of the patients opted for a tracheostomy and invasive ventilation. The clinical features are summarized in Table 1. For comparison, tissue was also collected from eight patients with sporadic ALS (sALS) and two patients heterozygous for a GGGGCC repeat expansion in *C9orf72* (*C9orf72HRE*). Similar tissues were obtained from 10 control patients of whom nine had died from other neurodegenerative conditions and one from a myocardial infarction (Supplementary Table 1, Online resource). To exclude the possibility of the patients also carrying mutations in other ALS-associated genes, screening for a panel of genes was performed. No pathogenic variants for ALS or FTD were found. Furthermore, DNA extracted from blood leucocytes and spinal cord tissue from all nine *SOD1*^*D90Ahom*^ patients are enrolled in ProjectMinE (www.projectmine.com). A number of whole-genome sequencing, genome-wide association, and DNA methylation studies are being performed within the ProjectMinE consortium. So far, no new variants of relevance to this report have been found (ongoing work). After being diagnosed with ALS, several of the patients also participated in clinical drug trials (riluzole, pyrimethamine) [31, 45] and neuropsychological, neurophysiological, and imaging studies (Table 1) [50, 51]. Following approval by the Ethics Committee and adhering to the Declaration of Helsinki with later amendments, written informed consent for DNA studies was obtained from each participant. This consent also includes permission to present and publish scientific results. With a separate approval from the Ethics Committee to perform research autopsies and save material for histological and molecular analysis, consent was obtained from the patients ante mortem and/or by consultation with next of kin post-mortem.

**Table 1:** Clinical features

Patient Number	1	2	3	4	5	6	7	8	9
Sex	Female	Male	Female	Male	Male	Female	Male	Female	Female
Age at onset^a^ (y)	38	58	53	38	55	38	51	54	50
Age at death^a^ (y)	43	66	64	53	70	55	75	81	82
Disease duration^a^ (y)	5	8	11	15	15	17	24	26	33
Pre-paresis symptoms	Lower back pain unsteadiness	Lower back pain	Lower back pain, severe myalgia	Lower back pain, instability left ankle	Lower back pain	Muscle cramps pain, heat sensations	Lower back pain	Lower back pain, paresthesia	Lower back pain
First paretic region	Legs (right to left)	Legs (left to right)	Legs (right to left)	Legs (left to right)	Legs (right to left)	Legs (left to right)	Legs (billat.)	Legs (right to left)	Legs (right to left)
Second motor region	Hand/arm (left to right)	Hand/arm (left to right)	Hand/arm (left to right)	Hand/arm	Hand/arm (right to left)	Hand/arm (left to right)	Hand/arm	Hand/arm (both at the same time)	Hand/arm (left to right)
Bulbar symptoms	Yes	Yes	Yes	Yes	Yes	Yes	Yes	Yes	Yes
Emotional lability	No	No	No	Yes	Yes	Yes	Yes	No	Yes
Urgency of micturition	No	Yes	Yes	Yes	No	No	Yes	Yes	No
Cause of death	Pneumonia	Pneumonia	Pulmonary embolism	Pneumonia	Pneumonia	Pneumonia	Pneumonia	Respiratory failure	Pneumonia
Participation in other studies^b^	MEP^40,41^	PET^21,22^ MEP^40,41^	PET^21,22^ MEP^40,41^	MEP^8^	PET^21,22^ MEP^40,41^	PET^21,22^ MEP^40,41^ Pyrimethamine^20^	PET^21,22^ MEP^8^ Riluzole^19^	ECAS	PET^21,22^ MEP^8,40,41^ Riluzole^19^ ECAS
RIG/PEG	Yes	Yes	Yes	Yes	No	Yes	Yes	Yes	Yes
NIV	Yes	Yes	Yes	Yes	Yes	Yes	Yes	Yes	No
Other relevant diseases	None	Hypertension, Psoriasis, Alcohol	Hypothyreosis, peptic ulcer	Hypertension DM type I Head trauma	Chronic peptic ulcer	None	Hypothyreosis, peptic ulcer	Hypertension DM Type II Atrial fibrillation	Migraine, Recurrent depressions
Tobacco smoking	Yes (heavy)	Yes (heavy)	Yes	Snuff (type of tobacco)	No	No	No	No	No
PM time (days)	2	2	2.5	2	1.5	1	1.5	3	2

Abbreviations used: *DM* diabetes mellitus, *ECAS* Edinburgh Cognitive and Behavioural ALS Screen, *NIV* non-invasive ventilation, *PEG* percutaneous endoscopic gastrostomy, *PET* positron emission tomography with either flumazenil (ref. [21]) or WAY100635 (ref. [22]) as ligands, *PM* post-mortem, *RIG* radiologically inserted gastrostomy, *MEP* transcranial magnetic motor-evoked potential (Refs. [8, 40, 41])

^a^Age and disease duration are presented in full years

^b^All nine patients participated in a broad range of studies. Some are listed here

#### Sampling of neurological tissue for histopathological studies

Tissue samples were collected at autopsy from defined areas according to a standard protocol [34] and immersion-fixed in 4% paraformaldehyde in 0.1 M Na phosphate, pH 7.4, at room temperature. After fixation for at least 6 weeks, CNS tissue were serially sectioned, and blocks from the following areas were dissected for further histological processing: From the frontal lobe, sections were taken from the precentral gyrus, the anterior cingulate gyrus, and the superior, middle, and inferior frontal gyri. From the temporal lobe, sections were taken from the amygdala, hippocampal formation (uncus and splenium), and lateral aspects of the temporal lobe. From the parietal lobe, sections were taken from the superior and inferior parietal lobule, including dorsal cingulate gyrus. From the occipital lobe, one section was taken from the primary visual cortex (Brodmann area 17). Sections from the deep grey nuclei were taken from the mid-portion of the caudate head, from the putamen (including the insular cortex), and from the thalamus at the level of the subthalamic nucleus. The brainstem was sampled by cross-sections, for the mesencephalon including the third cranial nerve, for the pons at a midlevel, and for the medulla at a midlevel of the olive. The spinal cord was sampled from the cervical, thoracic, lumbar, and sacral regions. Additionally, tissue samples were collected from 8 skeletal muscles, the myocardium, liver, kidney, spleen, lymph nodes, suprarenal glands, and skin.

#### Histochemistry and immunohistochemistry

Paraffin-embedded spinal cord sections (4 µm) were stained with haematoxylin and eosin and with Congo red according to standard protocols [7]. Sections from the nervous system were stained with luxol fast blue/cresyl violet using a modified Bielschowsky technique. In addition to haematoxylin and eosin staining, kidney and liver sections were stained using the Van Gieson technique, and for liver sections, the Perl’s, Laidlaw’s, and Fouchet’s techniques, as well as PAS and PAS-diastase staining, were performed according to established protocols [7].

For immunohistochemistry, paraffin-embedded spinal cord sections (4 µm) were immunostained according to the manufacturer’s recommendations using the ES system and ES reagents (Ventana Medical Systems Inc, Illkirch-Graffenstaden, France). The sections were preincubated for 30 min in 3% H_2_O_2_ in methanol and then heated in 0.5 M citrate buffer (pH 6.0) for 20 min in a microwave oven. To detect misfolded SOD1, polyclonal rabbit antibodies were raised in-house against keyhole limpet haemocyanin-coupled peptides. For this study, we chose to use two antibodies corresponding to amino acids (aa) residues 83–91 and 131–153, respectively, in the human SOD1 sequence. These antibodies have been evaluated using a range of dilutions (0.3–5 µg/ml for SOD1 aa131-153 ab; 2.3 µg/ml for SOD1 aa 83-91ab) and validated for specificity against misfolded SOD1 species. The high specificity of these antibodies has been validated earlier including in histological blocking assays with the immunization peptides [18, 20]. Additionally, here, we performed preincubation of the SOD1 aa 83–91 ab with the peptide used as immunogen (Supplementary Fig. 3a–c, Online resource). The small granular inclusions were only weakly detectable when the antibody was preincubated with an intermediate concentration of the immunizing peptide (0.1 µg/ml). No SOD1-positive inclusions were detected when the antibody was preincubated with a high concentration of the immunizing peptide (1.0 µg/ml) (Supplementary Fig. 3a–c, Online resource). Thus, both antibodies are well suited for immunohistochemical studies and bind only to misfolded SOD1 and not to natively folded SOD1. Immunohistochemistry was also performed with commercially available antibodies to hyperphosphorylated tau, ubiquitin, p62, pTDP-43, GFAP, Cystatin C, α-synuclein, amyloid-β, and slow myosin. (For a more detailed information on the antibodies, please see Supplementary Table 2, Online resource). The location of the primary antibodies was determined using corresponding fluorescent secondary antibodies or biotin-conjugated secondary antibodies coupled to an avidin-horseradish peroxidase conjugate. We used 3-amino-9-ethylcarbazole (brown colour) or Fast Red (red colour) as the precipitating enzyme product. Sections were counterstained with haematoxylin, washed, and mounted with Glycergel Mounting Medium (DakoCytomation).

As in previous studies [18–20], a four-point semiquantitative scale was used to score the number of neurons showing SOD1 inclusions in each section. The levels were 0 = no neurons with inclusions; 1 ≤ 25% of the neurons showing inclusions; 2 = 25–75% of the neurons showing inclusions; and 3 ≥ 75% of the neurons showing inclusions. The presence of SOD1 inclusions was rated independently by two examiners (KF, TB).

#### Immunolabelling for electron microscopy (immunoEM)

Tissues were fixed in 2% paraformaldehyde (PFA) (EM grade; Fisher Scientific, PA0095, batch no. 76336) and 0.2% glutaraldehyde (GA) (25% stock, EM grade; Taab Laboratories Equipment Ltd, G011/2, batch no. 24680) in 0.1 M phosphate buffer (PB), pH 7.4, at 4 °C overnight. The solution was replaced with freshly prepared 1% PFA in 0.1 M PB, pH 7.4, and stored at 4 °C. Fixed samples were washed three times in 0.1% glycine (Merck, 56-40-6) in phosphate-buffered saline (PBS) followed by incubation for 10 min at room temperature in 0.1% glycine in PBS. The solution was then replaced with 12% gelatine in 0.1 M PB, pH 7.4, and incubated at 37 °C for 30 min. Tissues were transferred to a fresh 12% gelatine solution and incubated for an additional 10 min at 37 °C and then placed on ice, cut out, and transferred to 2.3 M sucrose overnight at 4 °C. They were then mounted to metal pins facing the surface with an extra drop of 2.3 M sucrose to prevent ice crystal formation upon instant freezing in liquid nitrogen. To prepare cryosections, the samples were trimmed, and the presence of the tissue was confirmed under a light microscope. Ultrathin sectioning at a thickness of 70 nm was performed using an Ultra microtome (Leica EM UC7). Sections were placed on hexagonal copper grids with a formvar film and a carbon layer of approximately 3 nm. For immunogold labelling, sections were placed facing a 2% solution of 0.1 M PB, pH 7.4, for 5 min at room temperature and then for 20 min at 37 °C. The grids were washed three times for 2 min in 0.1% glycine in PBS, pH 7.4, and blocked for 5 min in 1% gelatine blocking buffer (1% gelatine from cold water fish [Sigma-Aldrich, G7041, lot # SLBN4365V] in PBS). The antibodies against misfolded SOD1, diluted in 1% gelatine blocking buffer, were added to the grids and incubated for 45 min. The grids were then washed five times in PBS with 0.1% gelatine blocking buffer for 2 min and incubated with 10 nm Protein-A-Gold (Cell Microscopy Core, University Medical Center Utrecht, The Netherlands) diluted 1:25–50 (batch dependent) in 1% gelatine blocking buffer for 20 min. The grids were then washed with PBS five times for 2 min. Fixation was performed with 1% glutaraldehyde in PBS for 5 min, followed by consecutive washes in deionized water. Contrasting was performed by incubating the grids in 2% uranyl oxaloacetate, pH 7. The grids were briefly washed in methyl-cellulose/uranyl acetate, pH 4, on ice and incubated for 5 min to avoid drying artefacts. The grids were looped out using in-house-made Remanium^®^ wire loops and imaged on a transmission electron microscope (Thermo Scientific Talos L120C TEM).

### Data availability

Reasonable data sharing requests are made in writing through the corresponding author (email) and require a formal data sharing agreement. Data sharing agreements must include details on how the data will be stored, who will have access to the data and intended use of the data, and agreements as to the allocation of intellectual property.

## Results

### Clinical findings

All patients described the onset of motor dysfunction as a sense of stiffness and complained of severe muscular cramps in the legs, unsteadiness or clumsiness, and general fatigue. A troublesome lower back pain and/or arthralgia of the hip or a knee region occurred in six of the nine patients in the months preceding the onset of first paresis. Radiological and arthroscopy examinations did not reveal any structural abnormalities explaining the pain. In eight patients, the paresis started asymmetrically in one of the legs, whereas patient #7 initially complained that both legs were “heavy as lead”. The paresis in all patients progressed to involve all extensor and flexor muscles in both legs before involving the muscles of the trunk and the upper extremities. Initial involvement of the arms was asymmetrical in all but one case where both arms were involved simultaneously. Bulbar symptoms appeared in all patients at, on average, 5.6 years (range 1–11 years) after the first symptoms in the legs. Dysarthria preceded dysphagia and dysphonia. Upper motor neuron signs (primarily brisk deep-tendon reflexes, Hoffmann’s sign, Babinski’s sign, and spasticity) preceded lower motor neuron signs when a new region (leg, arm, trunk, or bulbar) became involved. Upper motor neuron signs of involvement of a new region were often observed many months before symptoms appeared. Repeated clinical and neurophysiological examinations showed more prominent involvement of the legs than of the arms and the least involvement in the bulbar innervated muscles. One patient (#3) had prominent cerebellar ataxia, mainly in the arms, which appeared 3 years after the onset of the motor symptoms in the legs. Two patients also had reduced tactile sensory functions in the lower parts of the legs and in the feet. Loss of sense of vibration, first at the ankles and later also in the hands, was observed early in all patients. None of the patients showed overt signs of FTD, but five had prominent emotional lability in the later stages of the disease.

Patient age at onset of first symptom ranged from 38 to 60 years (mean 49 years) and duration of symptomatic illness ranged from 5 to 33 years (mean 17 years). The three patients with the shortest survival were all heavy cigarette smokers. Patients #3 and #5 both had a history of chronic ulcer of the stomach, and patient #4 was diagnosed with diabetes mellitus type 1 at the time the first paresis appeared.

#### Macroscopic neuropathological examination of *SOD1*^*D90Ahom*^ patients

The cerebral hemispheres did not show macroscopic signs of generalized atrophy. The brainstem and cerebellum appeared normal. Upon sectioning of the brain, no obvious pathology was found in the lenticular nucleus, the brainstem, or the cerebellum in any of the patients. The substantia nigra and locus coeruleus were well pigmented. The anterior roots of the spinal cord were atrophic in all nine patients.

#### Spinal cords of *SOD1*^*D90Ahom*^ patients show profound motor neuron loss

**Fig. 1:**
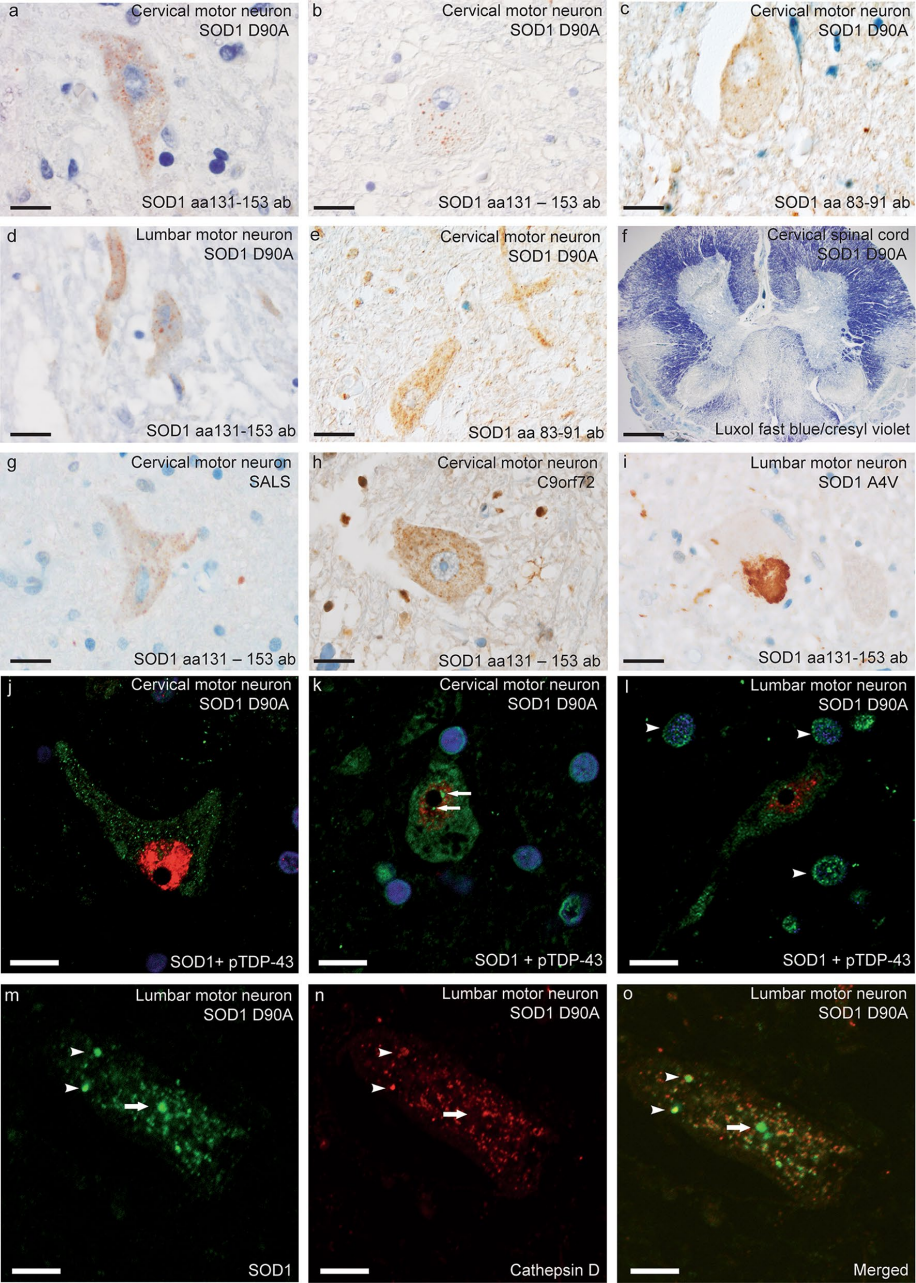
SOD1 staining in motor neurons and glial cells in spinal cord sections. Using the aa 83–91 and 131–153 anti-peptide antibodies against misfolded SOD1, numerous small granular SOD1 inclusions were observed in motor neurons in all nine *SOD1*^*D90A*^ patients (**a**–**e**). Myelin staining revealed severe degeneration of both the corticospinal tract and the dorsal column (**f**) in all patients. For comparison, small granular SOD1 inclusion were seen in a patient with sALS (**g**) and in an fALS patient with the *C9orf72HRE* mutation (**h**). Note the similar morphology of the SOD1 inclusions. Contrasting this, **i** a large skein-like inclusion containing mutated SOD1 was observed in a patient heterozygous for the *SOD1*^*A4V*^ mutation. In **j**–**o**, small granular immunopositive SOD1 inclusions are depicted in cervical (**j** and **k**) and lumbar (**l**, **m**) motor neurons stained with the aa 131–153 SOD1 anti-peptide antibody (green fluorescence). Both cytoplasmic staining (**j**) and intranuclear staining (**k**) can be seen in the motor neurons. In **l**, intranuclear and cytoplasmic SOD1 staining is apparent in a motor neuron surrounded by glial cells carrying intranuclear SOD1 staining (arrowheads). **j**–**l** were double-stained with TDP-43 (red fluorescence). Only nuclear and no cytoplasmic TDP-43 staining can be seen. In **m**, cytoplasmic SOD1 inclusions are seen, and in **n** lysosomes are visible using the lysosomal marker cathepsin D. In the merged picture (**o**), some SOD1 inclusions colocalize with the lysosomal marker (arrowheads), whereas others do not (arrow). Scale bar represent 20 µm in **a**–**c** and **g**–**i**, 40 µm in **d**, **e**, 960 µm in **f,** and 10 µm in **j**–**o**

Upon microscopic investigation, all nine patients had severe loss of motor neurons in the ventral horn to the point of rarefaction of the tissue and the appearance of cavities. There was a gradient where lumbar sections were more affected than cervical sections, and patients carrying the disease over 20 years had only a few definable lumbar motor neurons per section. In addition, the nuclei of Onufrowicz showed neuronal loss and gliosis. There was extensive demyelination of the lateral and the ventral corticospinal tracts. Staining with markers for gliosis, such as GFAP, showed positivity. Interestingly, the dorsal columns also had myelin and axonal loss to a similar degree (Fig. 1f). Massive neuronal degeneration was accompanied by intense gliosis in these tracts.

Using the aa 83–91 and 131–153 anti-peptide antibodies specific for misfolded SOD1 [20], small granular inclusions/aggregates were observed in the motor neuron somata of all patients at all levels of the spinal cord (Fig. 1a–e and j–m, Supplementary Fig. 3a, d, e Online resource). They measured 0.5–3 µm and were also seen in the neurons of Clarke’s nucleus. The D90A SOD1 inclusions had a morphology and distribution similar to the inclusions staining positive for misfolded wild-type SOD1 in sALS patients (Fig. 1g) [20] and in patients with mutations in other ALS- and FTD-associated genes (Fig. 1h) [19], but differ from the SOD1 staining seen in neurons from patients carrying dominantly inherited *SOD1* mutations, such as the *SOD1*^*A4Vhet*^ mutation. These patients carry larger skein-like inclusions (Fig. 1i and ref. [19]).

Noticeably, between 25 and 50% of all remaining motor neurons in each section had SOD1 inclusions. Also, in the two patients with the most severe motor neuron cell loss and muscle wasting (patients #8–9), small granular nuclear inclusions could be detected, even if only in a few motor neurons (Fig. 1e, j–l). The granular SOD1 inclusions partially colocalized with cathepsin D, a marker of lysosomes (Fig. 1m–o). Upon estimation, $\sim$ 20% of the cytoplasmic SOD1 inclusions colocalized with lysosomes (Fig. 1m–o). Nine of the control patients did not stain for SOD1 inclusions in the spinal cord or brain (Supplementary Fig. 1a, b, d–f, Online resource). A single patient diagnosed with epilepsy after a cerebral infarction had some SOD1 inclusions in the spinal cord and hypoglossal nucleus (Supplementary Fig. 1c, Online resource, described in detail in [19]).

In addition, misfolded intranuclear SOD1 was observed in glial cells of the affected spinal motor areas. Colocalization staining revealed that the majority of the cells were astrocytes, but oligodendrocytes and microglial cells were also present. The intranuclear glial SOD1 inclusions in different patients varied in frequency, affecting between 25 and 75% of the glial cells (Table 2).

**Table 2:** Summary of neuropathological findings in amyotrophic lateral sclerosis patients homozygous for the D90A *SOD1* mutation

Patient number	1		2		3		4		5		6		7		8		9	
NIA-AA, ABC score	A0B1C0, not		A2B0C1, low		A2B1C2, low		A0B0C0, not		A1B1C1, low		A0B0C0, not		A2B1C2, low		A3B2C2, intermediate		A2B2C2, intermediate	
SOD1 pathology	NCI	GI	NCI	GI	NCI	GI	NCI	GI	NCI	GI	NCI	GI	NCI	GI	NCI	GI	NCI	GI
Lower MN																		
* Cervical spin. cord*	+ + +	+ +	+ +	+	+ + +	+ +	+ +	+ +	+ +	+ +	+ +	+ + +	+ +	+	+ +	+ +	+ +	+ +
* Thoracic spin. cord*	+ + +	+ +	+ +	+	+ + +	+ +	+ +	+ +	+ +	+ +	+ +	+ + +	+ +	+	+ +	+ +	+ +	+ +
* Lumbar spin. cord*	+ + +	+ +	+ + +	+	+ + +	+ +	+ + +	+ + +	+ +	+ +	+ +	+ + +	+ + +	+ +	+ + +	+ +	+ +	+ +
* Sacral spin. cord*	n.a	n.a	+	+	+ + +	+ +	+ +	+ +	+ +	+ +	+ +	+ + +	+ +	+ +	+ +	+ +	+ +	+ +
Brain stem																		
* Substantia nigra*	+	+	0	+	0	+	+	+ +	0	+	0	+ + +	0	+	0	+	+	+
* Locus coerulius*	0	+	0	+	0	+	n.a	n.a	0	+	n.a	n.a	0	+	0	+	+	+
* Nucl. Pontine*	+ +	+	+	+	+ +	+ +	0	+ +	+	+	+ +	+ + +	+ +	+ +	+ +	+	+	+ +
* Nucl. Hypoglossus*	n.a	n.a	+	+	+ +	+ +	+ +	+ +	+ +	+ +	+ +	+ + +	+ +	+ +	+ +	+	+ + +	+ +
* N. vagus dorsalis*	n.a	n.a	0	+	+ +	+ +	+ +	+ +	+ +	+ +	+ +	+ + +	+ +	+ +	+	+	+ +	+ +
* Nucl. Ambiguus*	n.a	n.a	0	+	+ +	+ +	+ +	+ +	+	+ +	+ +	+ + +	+ +	+ +	+	+	+ +	+ +
* Nucl. Olivarius inf*	n.a	n.a	+ +	+ +	+	+	0	0	+	+ +	+ +	+ + +	+ +	+ +	+	+	+ +	+ +
Cerebral cortex																		
* Upper MN, Betz cells*	n.a	n.a	0	+	+	+	+ +	+ +	+	+ +	+	+ +	+ +	+	+	+ +	+	+ +
* Frontal lobe*	+	+	+	+	+	+	+	+	+	+ +	+	+ +	0	0	+	+	+	+
* Temporal lobe*	+	+	+	+	+ +	+	+	+	+	+ +	+	+ +	0	0	+	+ +	+	+
* Parietal lobe*	+	+	+	+	+ +	+	+	+	+ +	+ +	+	+ +	0	0	+	+	+	+
* Occipital lobe*	+	+	0	+	+	+ +	+	+	+	+ +	+	+ +	0	0	+	+	+	+
Hippocampus	+ +	+	0	0	+ +	+ +	+	+	+	+	+	+ + +	+	+	+	+	+ +	+ +
Clark’s nucleus	+ + +	+ +	+ +	+	+ + +	+ +	+ +	+ +	+	+	+ +	+ + +	+	+ +	+ +	+ +	+ +	+ +
Dentate nucleus	+	+	0	+	+	+ +	0	+	0	+	+ +	+ + +	+ +	+ +	+ +	+ +	+	+
Cerebellar cortex	+	+	0	+	+	+ +	0	+	0	+	0	+ +	+ +	+ +	+	+ +	+	+
Thalamus	n.a	n.a	+	+	+	+	+	+ +	+	+	+ +	+ +	n.a	n.a	+ +	+ +	+ +	+ +
Basal ganglia	0	+	+	+	+	+	+	+ +	+	+	+ +	+ +	+	+	+ +	+ +	+ +	+ +

Abbreviations: case—case number; NIA-AA, ABC score—National Institute on Ageing-Alzheimer’s Association, A = A score converted from Thal beta-amyloid Phase, B = B score converted from Braak and Braak NFT stage, C = CERAD (Consortium to Establish a Registry for Alzheimer’s Disease) neuritic plaque score [36]; *MN* motor neurons, *SOD1* semiquantitatively assessed using anti-misSOD1 immunohistochemistry as follows: 0 none, not detectable, + mild (< 25% neurons stain), +  + moderate (25–75% neurons stain), +  +  + severe/numerous (> 75% neurons stain); *NCI* Neuronal cytoplasmic inclusion, *GI* Glial inclusions, *n.a.* not available

Similar to what has been reported in ALS patients lacking *SOD1* mutations, small granular inclusions containing cystatin C were seen in some motor neurons and neurons of Clarke’s column in all nine patients. Bunina bodies could not be detected in the remaining motor neurons in the spinal cord.

#### Patients with the ***SOD1***^***D90Ahom***^ mutation have widespread brainstem pathology

**Fig. 2:**
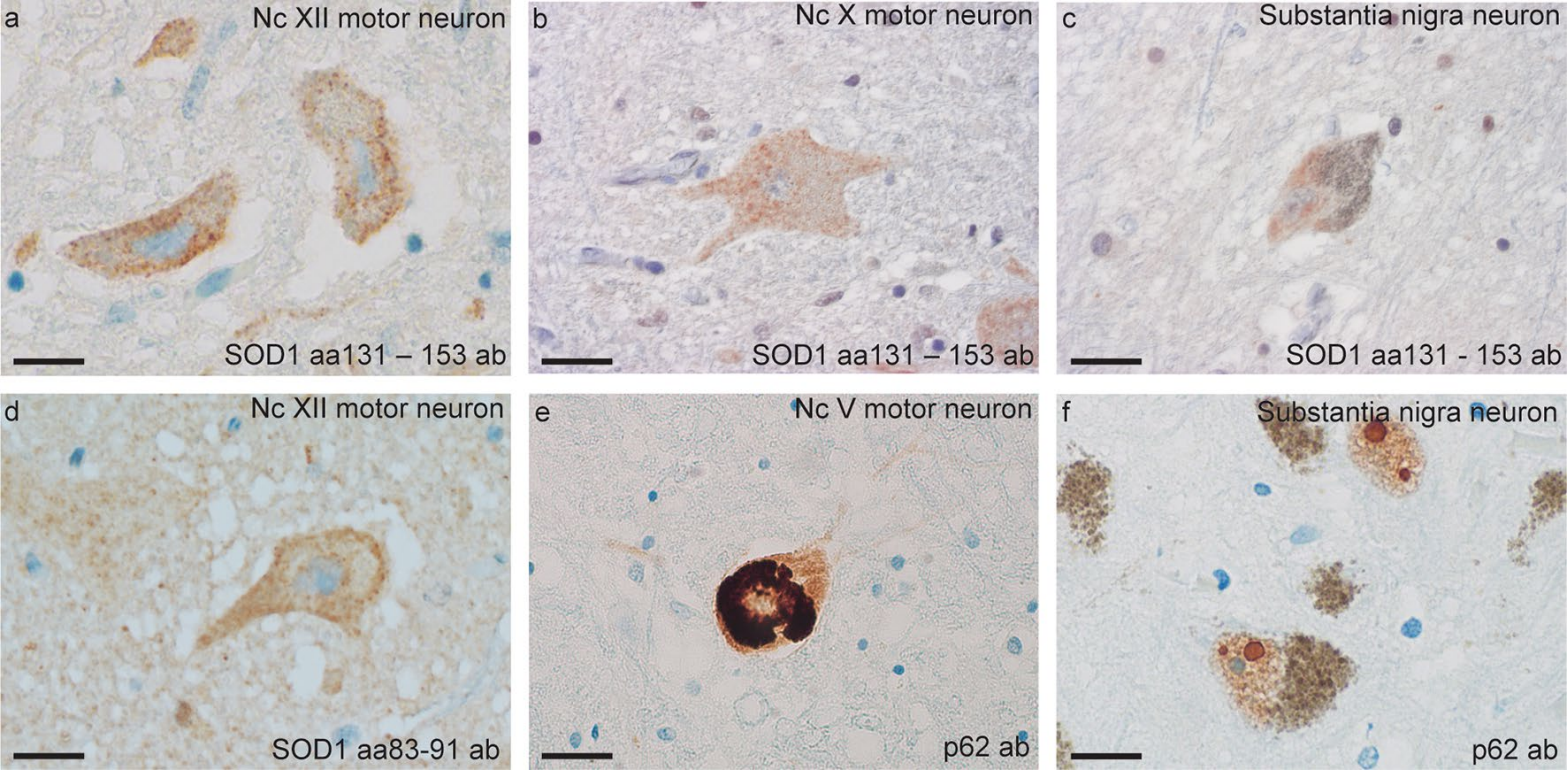
SOD1 and p62 staining in brainstem nuclei. Using the aa 83–91 and 131–153 anti-SOD1 peptide antibodies recognizing misfolded SOD1, numerous small granular inclusions were found in the hypoglossal nuclei in all nine *SOD1*^*D90Ahom*^ patients (**a** and **d**). Inclusions were also seen in the dorsal motor nucleus of the vagus nerve, but to a lesser extent (**b**). Representative pictures are shown. In two patients, larger inclusions were seen in neurons of the dorsolateral substantia nigra (**c**). The aa 83–91 anti-SOD1 peptide antibody detected intraneuronal small granular inclusions with the same morphology as those detected by the aa 131–153 anti-SOD1 peptide antibody (**d**). p62 immunohistochemistry revealed large cytoplasmic inclusions in motor neurons of the trigeminal nucleus (**e**). Marinesco bodies were frequently found in the nuclei of substantia nigra neurons (**f**). Scale bar represent 18 µm

In all *SOD1*^*D90Ahom*^ patients, brainstem motor neurons showed the same type of small granular SOD1 inclusion as described above (Fig. 2a–b and d). The hypoglossal nuclei were severely affected by cell loss and SOD1 inclusions were seen in $\sim$ 75% of the remaining motor neurons. The motor neurons of the facial motor nuclei also had SOD1 inclusions but to a slightly lesser extent than the hypoglossal nuclei ($\sim$ 50% upon estimation). In the two patients with the most pronounced loss of spinal motor neurons, larger conglomerate SOD1-positive inclusions were seen in pigmented neurons of the caudal–dorsal parts of the substantia nigra (Fig. 2c) and in the oculomotor and ambiguus nuclei.

**Fig. 3:**
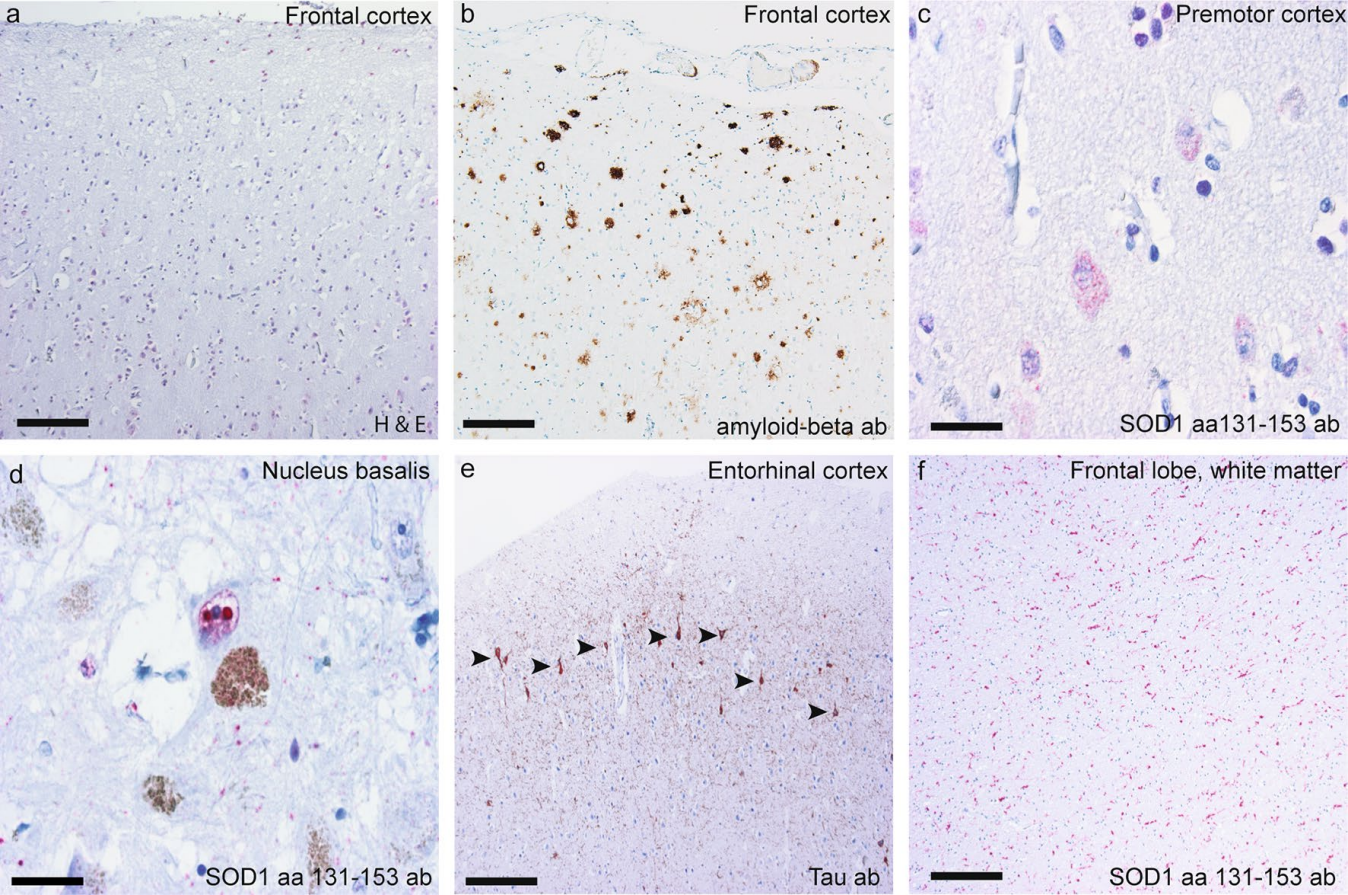
Micrographs depicting brain and brainstem pathology in *SOD1*^*D90Ahom*^ patients. **a** Frontal premotor cortex showing morphological signs of microvacuolation. Note the vacuoles surrounding the cells in laminae I–III. **b** Amyloid beta staining of the frontal cortex showing neuritic plaques. **c** Cytoplasmic SOD1 staining in Betz cells of the premotor cortex stained with the aa 131–153 anti-peptide SOD1 antibody. **d** Intranuclear Marinesco bodies in neurons of the nucleus basalis. **e**. Tau staining of the entorhinal cortex. The arrowheads show intraneuronal tangles. **f** Neurites in the cortical white substance stained with the SOD1 anti-peptide antibody. Scale bars represent 50 µm (**a**), 60 µm (**b**, **e** and **f**), 40 µm (**c**), and 20 µm (**d**)

Ubiquitin and p62 staining revealed a few granular cytoplasmic inclusions in trigeminal motor neurons (Fig. 2e), and intranuclear Marinesco-like bodies were frequently seen in the neurons of substantia nigra (Fig. 2f) and nucleus basalis. Some of these were positive for misfolded SOD1 (Fig. 3d). The amygdala had pathological changes with gliosis and neuronal loss. Staining for ubiquitin and p62 revealed intracellular globules and neurites in the entorhinal and frontotemporal region. Some of these also stained positive for amyloid beta (Fig. 3b).

**Fig. 4:**
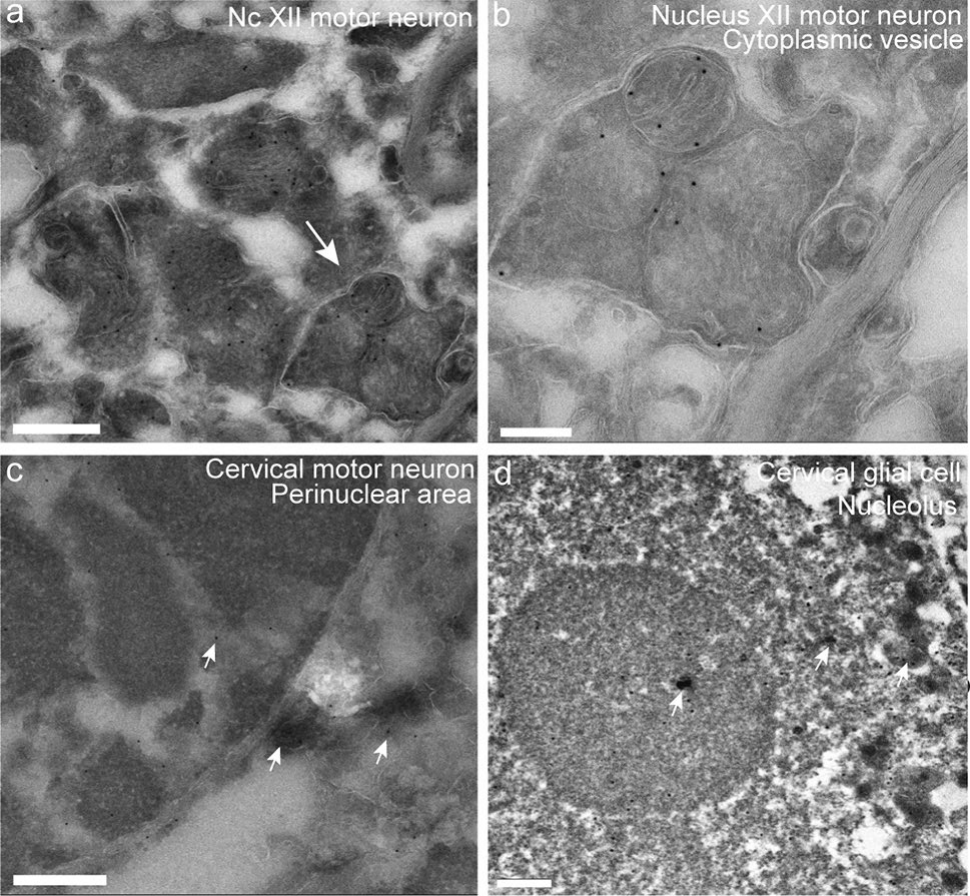
Immunoelectron micrographs of *SOD1*^*D90Ahom*^ patient #9 showing positive staining for misfolded SOD1 localized in degenerated mitochondria, ER, and the nuclei of motor neurons and glial cells. All sections were stained with the aa 131–153 anti-SOD1 peptide antibody. **a** Degenerated mitochondria and endoplasmic reticulum stained with immunogold-labelled aa 131–153 anti-SOD1 peptide antibody in a hypoglossal nucleus motor neuron. Note the vesicle containing mitochondria and misfolded SOD1^D90A^. **b** Higher magnification of the vesicle depicted in **a**. In **c**, the nucleus of a cervical spinal cord motor neuron is depicted. Note the immunogold-labelled misfolded SOD1^D90A^ in the nucleus and perinuclear area. **d** A glial cell nucleus with immunogold-labelled misfolded SOD1^D90A^. Both the nucleolus and the nucleus stain positive for misfolded SOD1^D90A^. Scale bars represent 500 nm in (**a**–**c**) and 200 nm in **b**

From patient #9, tissue was saved for ImmunoEM (IEM) at autopsy. Sections were taken from the hypoglossal nuclei and the ventral horn of the lumbar and cervical spinal cord and stained with the SOD1 aa 131–153 antibody. At the ultrastructural level, lumbar sections revealed severe neurodegeneration, and no representative tissue from motor neurons could be investigated. However, motor neurons could be identified at the cervical level and in the hypoglossal nuclei. Cytoplasmic staining of misfolded SOD1 was found in degenerated mitochondrial structures and in the region close to the nucleus (Fig. 4). Some mitochondrial structures were identified in vesicles that were scattered in the cytoplasm (Fig. 4a, b). Some positive staining for SOD1^D90A^ was seen in both the nucleus as well as in the perinuclear area (Fig. 4c). For glial cells, staining for SOD1 was occasionally observed in the nuclei (Fig. 4d).

#### Concomitant pathology in ***SOD1***^***D90Ahom***^ patients

Staining for phosphorylated pTDP-43 in *SOD1*^*D90Ahom*^ patients was mostly negative and did not reveal pathological cytoplasmic staining (Supplementary Table 3, Online resource). The exception was patient #3, in whom a single positive pTDP-43 inclusion in one motor neuron in one section of the cervical spinal cord was observed (Supplementary Fig. 2a, Online resource). This patient and two more patients (patients #5 and #6) also had some pTDP43 staining of the neuropil in the dorsal horn lamina I-IV (Supplementary Fig. 2b, Online resource). For comparison, the eight sALS patients and the two patients carrying a *C9orf72HRE* mutation had numerous pathological pTDP-43 inclusions in the spinal cord and hypoglossal nuclei, including fine skeins, coarse skeins, dot-like and dense round inclusions (Supplementary Fig. 2c, d, Online resource). Staining with antibodies against FUS and alpha-synuclein did not show any aberrant CNS findings in any patient.

ABC staging [36] was performed using antibodies against hyperphosphorylated PFH-Tau and amyloid beta (Table 2). A few senile plaques and neurofibrillary tangles were observed in four patients (patients #3 and #7–9) (Fig. 3e), but these were insufficient to warrant a diagnosis of Alzheimer’s disease. Some of the senile plaques stained positive for SOD1 (Supplementary Fig. 2e, Online resource). When double staining was performed with an antibody against amyloid-β, colocalization was observed (Supplementary Fig. 2f, Online resource). This is a known finding in ALS patients and patients with Alzheimer’s or Parkinson’s diseases [13].

#### Cortical pathology in the ***SOD1***^***D90Ahom***^ patients

**Fig. 5:**
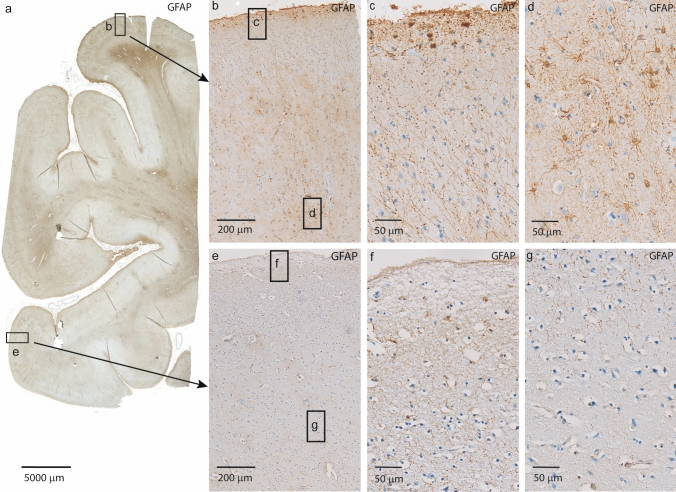
GFAP staining in the temporal lobe showing prominent and focally increased gliosis in a *SOD1*^*D90Ahom*^ patient. Note the areas of focally increased gliosis. In **b**–**d**, heavy gliotic staining is seen that involves all layers and is also seen in the subcortical white matter, as opposed to **e**–**g**, where only sparse glial staining is seen that does not affect all layers. The areas with focally increased gliosis were seen in all nine *SOD1*^*D90Ahom*^ patients. They were widespread and affected all cortical areas, including the temporal, frontal, and parietal lobes

The cortical histopathology is described below and summarized in Table 2. Mild cortical atrophy with neuronal loss, reactive astrocytosis and microvacuolization of the three superficial cortical laminae was observed (Fig. 3a) in all patients along with pathological gliosis. In the frontal lobe, the gliosis was widespread and seen in all cortical laminae but appeared focally in the temporal and parietal lobes, where superficial lamina I was most affected (Fig. 5). In focal areas with marked increased gliosis, intranuclear SOD1-positive inclusions in glial cells were observed. Some neurons had more diffuse cytoplasmic SOD1 staining (Fig. 3c). No obvious correlation between the degree of gliosis and survival time or age was observed.

Surprisingly, the most conspicuous finding was neurites positively stained for misfolded SOD1 in the deep white matter of the frontal and anterior temporal lobes and the insula, accompanied by marked gliosis (Fig. 3f). Furthermore, p62 staining revealed an extracellular globular and neuritic pattern that dominated the histopathological picture in layer II in the frontal and temporal lobes and in the entorhinal cortex. Intracellular inclusions were only rarely observed. All patients had ubiquitin and p62-positive globules and neurites in the entorhinal cortex, and six patients also had globules and neurite staining in the CA4 areas. In one patient (#3), the whole hippocampal formation was affected. Altogether, these findings are sufficient to warrant a histopathological diagnosis of frontotemporal lobar degeneration (FTLD).

#### Pathology of muscles and peripheral organs

**Fig. 6:**
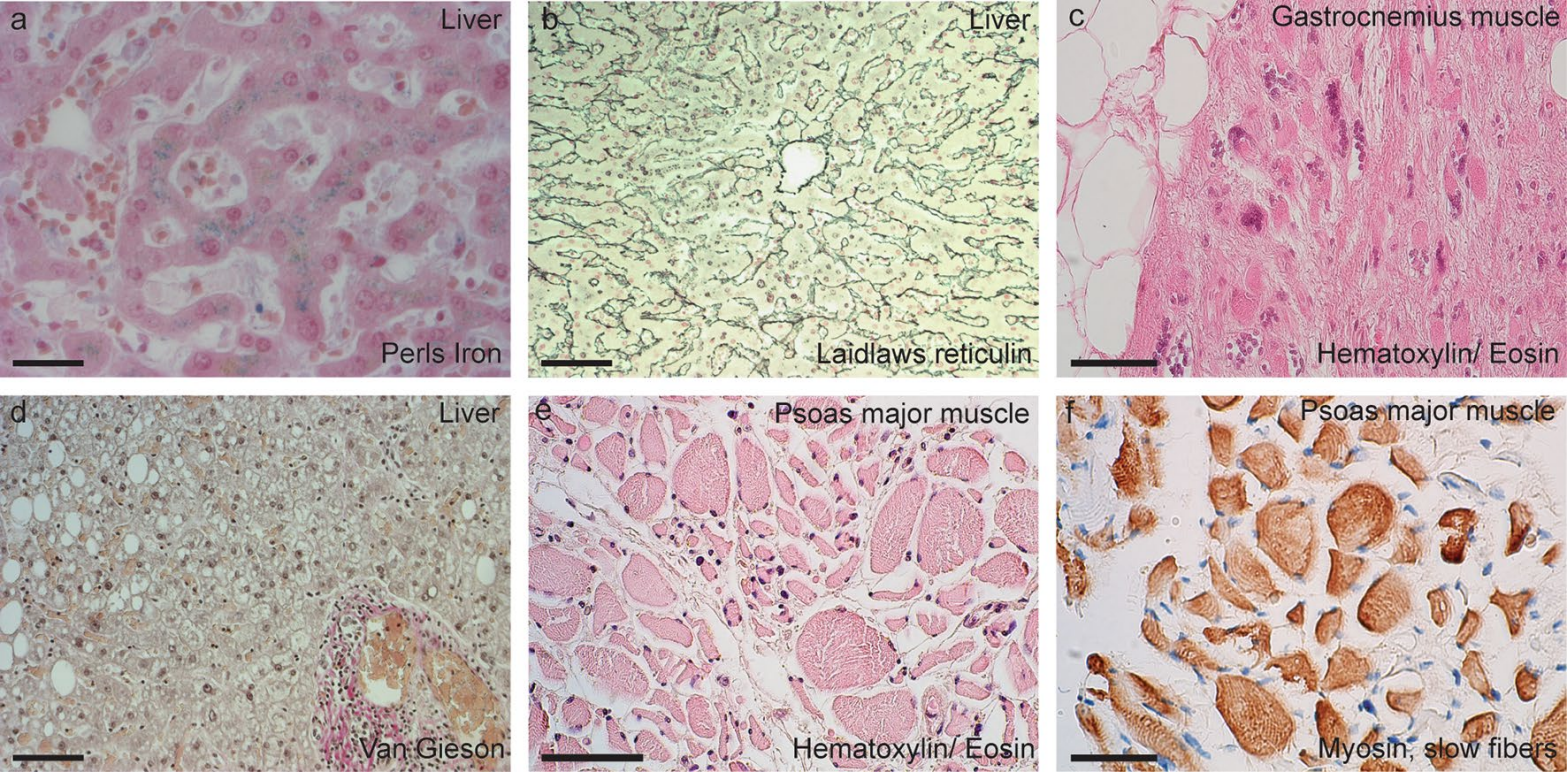
Micrographs of liver and muscle tissue. Accumulation of iron in liver cells was seen in three of the nine patients (**a**) (Perl’s stain). One of these patients showed normal morphology of connective tissue in the liver (**b**) (Laidlaw’s reticulin stain), while in another patient, steatosis and a mild periportal inflammatory cellular infiltrate composed of lymphoplasmocytes were seen (**d**) (Van Gieson stain). In Panels **c**, **e,** and **f**, neurogenic atrophy of gastrocnemius and psoas muscle is shown. In panel f, the muscle is stained with an antibody against myosin, staining slow muscle fibers. Note the angular shape of the muscle fibers and the grouping of atrophied fibers. The scale bars represent 18 µm in (**a**), 60 µm (**b**, **d**–**f**), and 100 µm in (**c**)

Skeletal muscles showed neurogenic group atrophy in the upper and lower limb and in the trunk and neck muscles of all patients (Fig. 6c and e). When stained with an antibody against myosin slow fibers, neurogenic grouping of atrophied fibers was shown (Fig. 6f). The degeneration was most severe in lower limb muscles but was also present in upper limb muscles as well as in the tongue. No pathological changes were seen in the kidney or the heart. In three patients, an accumulation of iron in hepatocytes and Kupffer cells was observed (Fig. 6a). There were no signs of cirrhosis (Fig. 6b and d), and testing for the p.H63D and p.C282Y mutations in *HFE* was negative. Two patients had steatosis, and periportal infiltration of lymphoplasmocytes was observed in one patient (Fig. 6d). Staining for misfolded SOD1 was negative in myocardium, muscle, kidney, and liver tissue.

## Discussion

ALS is now recognized as a syndrome heterogenous in aetiology, clinical presentation, genetic variants, neuropathology, and prognosis. Here, we report the neuropathological findings in nine patients homozygous for the *SOD1*^*D90A*^ mutation. Using anti-peptide antibodies with high specificity for misfolded forms of the SOD1 protein [20], we found small granular cytoplasmic inclusions of mutant SOD1 in motor neurons in the brain and spinal cord in all nine patients. SOD1-containing inclusions are hallmarks of ALS caused by *SOD1* mutations [10, 23, 27, 43, 48], but patients heterozygous for dominantly inherited ALS-causing *SOD1* mutations, e.g., *SOD1*^*G127X*^ and *SOD1*^*A4V*^, have larger skein-like SOD1-containing inclusions (Fig. 1j) [23, 47].

As in ALS in general, symptomatic disease in *SOD1*^*D90Ahom*^ patients present with focal onset with subsequent spread along neuroanatomical connections and to adjacent areas. SOD1 aggregates prepared from transgenic mice overexpressing human *SOD1* mutations or from an *SOD1*^*G127Xhet*^ fALS patient, upon inoculation into the spinal cord of 100 day old mice result in propagation of SOD1 aggregation almost exclusively in the motor system with concomitant development of an ALS-like phenotype [8, 15]. Speculatively, such a prion-like mechanism could be part of the pathomechanism in SOD1-provoked ALS and be mediated by the granular inclusions in *SOD1*^*D90Ahom*^ patients described herein.

While most mutations in *SOD1* give rise to proteins that are structurally unstable, the SOD1^D90A^ mutant protein is structurally stable and has enzymatic activity that is as high as wild-type SOD1 [33]. In erythrocytes and in the CNS of *SOD1*^*D90Ahom*^ patients, SOD1 activity is over 90% of that of controls [4, 24]. Thus, the SOD1^D90A^ mutant protein appears to be a link between other ALS-associated mutant SOD1s and the wild-type protein. In a comparison of the pathomorphology of SOD1 inclusions in *SOD1*^*D90Ahom*^ patients with the SOD1 inclusions observed in sALS and fALS patients lacking *SOD1* mutations [19, 20], the similarities and differences can be summarized as follows: At the cellular level, we found no differences in the morphology and pattern of cellular distribution of SOD1-positive inclusions in sALS patients (Fig. 1g) compared to *SOD1*^*D90Ahom*^ patients, and the number of inclusions in individual motor neurons was of the same magnitude (Fig. 1a–e, j–m). Furthermore, the SOD1^D90A^ inclusions partially colocalized with a lysosomal marker in a similar way to SOD1 inclusions in the patients lacking *SOD1* mutations (Fig. 1m–o). Also, the number of spinal motor neurons containing granular inclusions was higher in ALS patients lacking *SOD1* mutations than in the *SOD1*^*D90Ahom*^ patients, where fewer pyknotic and damaged neurons remained. This finding may be explained by the far longer survival time of the *SOD1*^*D90Ahom*^ patients, resulting in a more severe loss of neurons. Finally, the number of remaining motor neurons is less than normally seen in ALS patients lacking *SOD1* mutations. Thus, comparison of proportion of nerve cells having SOD1 inclusions should be made with caution. Consistent with our current and previous observations [19, 20], it was recently reported that impaired maturation, reduced specific activity, and increases in post-translational modifications in SOD1 are regularly found not only in patients with *SOD1* mutations but also in other fALS and sALS [49]. In accord with a previous observation in patients lacking *SOD1* mutations [18], there were intranuclear SOD1-positive inclusions in glial cells, indicating neuroglial involvement to various extents (Table 2).

Patients with pathogenic mutations in *SOD1* and *FUS* are neuropathologically distinct from other types of ALS with the absence of cytosolic pTDP-43 proteinopathy [32]. Two case reports have described cytoplasmic pTDP-43 proteinopathy in patients with *SOD1* mutations [17, 37]. Interestingly, one of these was a patient with bulbar-onset ALS *heterozygous* for the D90A *SOD1* mutation [17] with typical pTDP-43-positive neuropathology, supporting the hypothesis that the pathomechanism may be different in (some) D90A-heterozygous patients compared to *SOD1*^*D90Ahom*^ patients. The patient reported by Feneberg et al. [17] had a phenotype and short disease course different from all reported *SOD1*^*D90Ahom*^ patients. All nine *SOD1*^*D90Ahom*^ patients stained negative for cytoplasmic pTDP-43 proteinopathy except for patient #3, who had a minute amount of pTDP-43 staining in a single motor neuron in the cervical spinal cord (Supplementary Fig. 2a, Online resource). In addition, the same patient and also patients #5 and #6 had pTDP-43staining confined to the dorsal horns (Supplementary Fig. 2b, Online resource). The relevance of this pTDP-43 staining is not known.

The neuropathological findings were remarkably similar in all nine patients and closely matched the observed uniform clinical signs and test results from neurophysiological and imaging studies. An early MEP finding in *SOD1*^*D90Ahom*^ patients is delayed central motor conduction latency reminiscent of what is observed in a demyelinating disease rather than an axonal condition [3, 52, 53]. Frequently, in the first years after symptom onset, aberrant MEP results are a more prominent feature than findings on EMG examinations, suggesting that early UMN-axonal involvement is a key event in this type of ALS. This is corroborated by the extensive damage to the pyramidal tracts observed in all nine cases. Similarly, central sensory-evoked potential testing may be pathological, and this is reflected in the lesions observed to the posterior columns (Fig. 1f). Combining the earlier reported clinical and neurophysiological findings, we speculate that the uniform syndrome observed in *SOD1*^*D90Ahom*^ patients is a condition initially affecting the longest and hence most vulnerable tracts. Six of the patients participated in PET studies (Table 1) [50, 51]. Using the GABA_(A)_ receptor ligand flumazenil, decreases in binding were observed in both motor and non-motor areas in the frontotemporal and anterior cingulate cortices [50]. PET with the 5-HT_(1A)_ receptor ligand WAY100635 showed involvement of all cortical areas, which was most pronounced in the left anterior lateral temporal lobe and Broca’s area [51]. Interestingly, in two presymptomatic *SOD1*^*D90Ahom*^ subjects who participated in the PET studies, a small focus with the most pronounced involvement was the left anterolateral temporal region, suggesting that this part of the CNS is among the earliest to be involved [51]. Cortical histopathology was apparent and in line with the findings of PET studies in *SOD1*^*D90Ahom*^ (Figs. 3a–f and 5) (Table 2) [50]. Some of the findings resemble the pathology seen in patients with frontotemporal lobar degeneration (FTLD) with astrocytic gliosis, microvacuolization, and swollen neurons that stain positive for ubiquitin and p62. Clinical FTD share many molecular and genetic aspects with ALS. Mutations in *C9orf72HRE*, *progranulin*, *FUS, TBK1, VCP, ANG, NEK1,* and *TARDBP* are pleiotropic and recognized causes of both ALS and FTD [21, 26, 46]. The nine *SOD1*^*D90Ahom*^ patients tested negative for mutations in these genes. Although overt cognitive dysfunction is less prominent in ALS patients with *SOD1* mutations [54], rare patients heterozygous for *SOD1* mutations with Broca’s aphasia as the first symptom of FTD-ALS disease have been reported [28]. Five of the nine ALS patients reported here had affective lability, but clinically significant frontal lobe dysfunction (requiring medication) were never observed. Since 1994, we have performed neuropsychological testing for frontal lobe function using a variety of psychological tests, including the Edinburgh Cognitive and Behavioural ALS Screen (ECAS). The here autopsied *SOD1*^*D90Ahom*^ patients participated in these studies. We found that all *SOD1*^*D90Ahom*^ patients had some degree of cognitive impairment that was most pronounced in the linguistic and executive domains (manuscripts in preparation), [50, 51] that was not apparent in a clinical setting. We speculate that the slowly developing pathology and long survival time in *SOD1*^*D90Ahom*^ patients leaves sufficient time for other CNS areas to compensate before becoming affected to a clinically detectable level. This hypothesis is supported by the observation that patients with other *SOD1* mutations and more typical ALS disease when placed on invasive ventilation treatment for years develop widespread pathology [35].

This is the first neuropathological study of patients homozygous for an essential free radical scavenging enzyme. The essentially normal enzymatic activity protects *SOD1*^*D90Ahom*^ patients from developing superoxide-free radical-induced damage. Nine other *SOD1* mutations have in rare cases been reported in homozygous form, most notably the recently discovered p.C112Wfs*11 and p.V120delV *SOD1* in children [5, 16, 38]. These mutations result in SOD1 proteins devoid of enzymatic activity and the children already have onset in infancy of a more generalized neuronal phenotype, but strikingly—as in the *SOD1*^*D90Ahom*^ patients—the first apparent symptoms are from the motor system with early involvement of the UMN system. These children also develop signs from some other organ systems; the most plausible explanation is the absence of SOD1 enzymatic activity [5, 16, 38]. In none of the nine *SOD1*^*D90Ahom*^ did we observe pathology outside the CNS attributable to the *SOD1*^*D90Ahom*^ genotype, even in the patients who had a symptomatic motor neuron disease for over 20 years. This is consistent with findings in human *SOD1* transgenic rodents, where no pathology is found in peripheral organs in mice that develop paralysis spontaneously or following spinal SOD1 aggregate inoculations. Even inoculation of copious amounts of SOD1-containing aggregates outside the CNS fails to induce pathology to peripheral organs [29].

In summary, we found that patients carrying the *SOD1*^*D90Ahom*^ genotype have multiple small granular inclusions of misfolded SOD1 in the cytoplasm of motor neurons. Furthermore, there is severe degeneration of the motor and sensory long tracts in the spinal cord, extensive neuronal loss in the spinal cord, and widespread cortical pathology with neurodegeneration and focal gliosis in frontotemporal cortical areas. Thus, these findings are in line with evidence from genetic, neurophysiological, neuropsychological, and clinical examinations that the pathological process in the slowly evolving *SOD1*^*D90Ahom*^ disease is not confined to the motor system but rather is a more generalized neurological syndrome affecting specific parts of the nervous system.

## Supplementary Information

Below is the link to the electronic supplementary material.

Supplementary file1 (PDF 2444 KB)

## Abbreviations

AA: Amino acids
ALS: Amyotrophic lateral sclerosis
CNS: Central nervous system
*C9orf72HRE*: Hexanucleotide repeat expansion in *C9orf72*
ECAS: Edinburgh Cognitive and Behavioural ALS Screen
fALS: Familial amyotrophic lateral sclerosis
FTLD: Frontotemporal lobar dementia
FUS: Fused in sarcoma
HFE: Hemochromatosis gene
MEP: Motor-evoked potential from transcranial magnetic stimulation of the motor cortex
PB: Phosphate buffer
PBS: Phosphate buffered saline
pTDP-43: Phosphorylated transactive response DNA-binding protein 43
sALS: Sporadic amyotrophic lateral sclerosis
SOD1: Superoxide dismutase-1
