# Supplementary material for: Widespread CNS pathology in amyotrophic lateral sclerosis homozygous for the D90A SOD1 mutation
Source: Acta Neuropathol. 2022 Nov 16;145(1):13–28. doi: 10.1007/s00401-022-02519-z (PMC9807479; doi:10.1007/s00401-022-02519-z)
Supplement: Supplementary file 1 — Supplementary file1 (PDF 2444 KB) [file 401_2022_2519_MOESM1_ESM.pdf]

Supplementary Information to

Widespread CNS pathology in amyotrophic lateral sclerosis homozygous for the D90A *SOD1* mutation

Karin M. Forsberg[1,2] , Karin S. Graffmo[2] , Erica Stenvall[2], Naima Tabikh[2],
Stefan L. Marklund[3], Thomas Brännström[2], Peter M. Andersen[1*]

Affiliations:

[1] Department of Clinical Sciences, Neurosciences, Umeå University, Umeå, Sweden

[2] Department of Medical Biosciences, Pathology, Umeå University, Umeå, Sweden

[3] Department of Medical Biosciences, Clinical Chemistry, Umeå University, Umeå, Sweden

*Corresponding Author:
Peter Munch Andersen
Department of Clinical Science, Neurosciences, Umeå University, SE-901 85 Umeå, Sweden.
Email: peter.andersen@umu.se
Mobile: +46 70 647 6913
ORCID# 0000-0003-0094-5429

Content:

1. Supplementary Figure 1: Staining for misfolded SOD1 in control individuals.

2. Supplementary Figure 2: Staining for pTDP43 and beta-amyloid.

3. Supplementary Figure 3: Blocking experiment using the SOD1 aa 83-91 peptide

4. Supplementary Table 1: Demographics of controls and other ALS patients

5. Supplementary Table 2: List of antibodies used

6. Supplementary Table 3: Grading of pTDP43 staining in ALS patients

Legends to Supplementary Figure 1

Spinal cord and medulla oblongata sections of control patients with other neurodegenerative diseases and non-neurological conditions. **a** Spinal cord motor neurons from a patient who died of myocardial infarction (C #7), **b** spinal cord motor neurons from a patient with PD (C #2), **c** spinal cord motor neurons from a patient who died from status epilepticus after stroke (C #4), **d** spinal cord motor neurons from a patient who died from Parkinson's Disease (PD) (C #9), **e** neurons of the hypoglossal nucleus from a patient who died from PD (C #10), **f** neurons of the hypoglossal nucleus from a patient with Alzheimer's Disease (C #1). All sections were stained with the aa131-153 SOD1 peptide antibody. Panels (**a-b**, **d-f**) stained negative for the aa131-153 SOD1 antibody. In panel **c** multiple small granular cytoplasmic inclusions of misfolded wtSOD1 can be seen in motor neurons with a size of approximately 0.5 - 3 µm. Scale bars **a-h** 20 µm

Legends to Supplementary Figure 2

a-d Spinal cord sections showing cervical motor neurons stained with an antibody against pTDP43. **a-b** are from $SOD1^{D90Ahom}$ patient (#3) and **c-d** from patients with sporadic ALS (S#8 and S#3, respectively). A single skein-like inclusion positive for pTDP43 was found in one section in the $SOD1^{D90Ahom}$ patient (**a**). pTDP43 staining was also seen in the dorsal horn (**b**). In contrast numerous skein and dot-like granular pTDP43 inclusions were found in all 8 sALS patients (**c-d**). (**e-f**) show a senile plaque in the motor cortex from $SOD1^{D90Ahom}$ patient (#8). Using the aa131–153 anti-peptide antibodies against misfolded SOD1 some of the senile plaques stained positive for SOD1 (**e**). When double staining was performed with an antibody against amyloid-β, colocalization was observed (**f**). Scale bars **a-d** 20 µm **e-f** 50 µm

Legends to Supplementary Figure 3

Micrographs showing the effect of preincubation of the primary SOD1 aa 83-91 antibody with the peptide used as immunogen (**a-c**) and SOD1 immunoreactive inclusions in spinal cord motor neurons (**d-e).** Several small granular inclusions in the soma were seen when the antibody was preincubated only with diluent (**a**). The small granular inclusions were only weakly detectable when the antibody was preincubated with an intermediate concentration of the immunizing peptide (0.1 µg/ml) (**b**). No SOD1-positive inclusions were detected when the antibody was preincubated with a high concentration of the immunizing peptide (1.0 µg/ml) (**c**). Several small SOD1 inclusions were seen in the cytoplasm of spinal cord motor neurons using the SOD1 aa 83-91 antibody both with immunohistochemistry (d) and immunofluorescence (e). Scale bars represent 20 µm in **a-e**.

Supplementary Figure 1

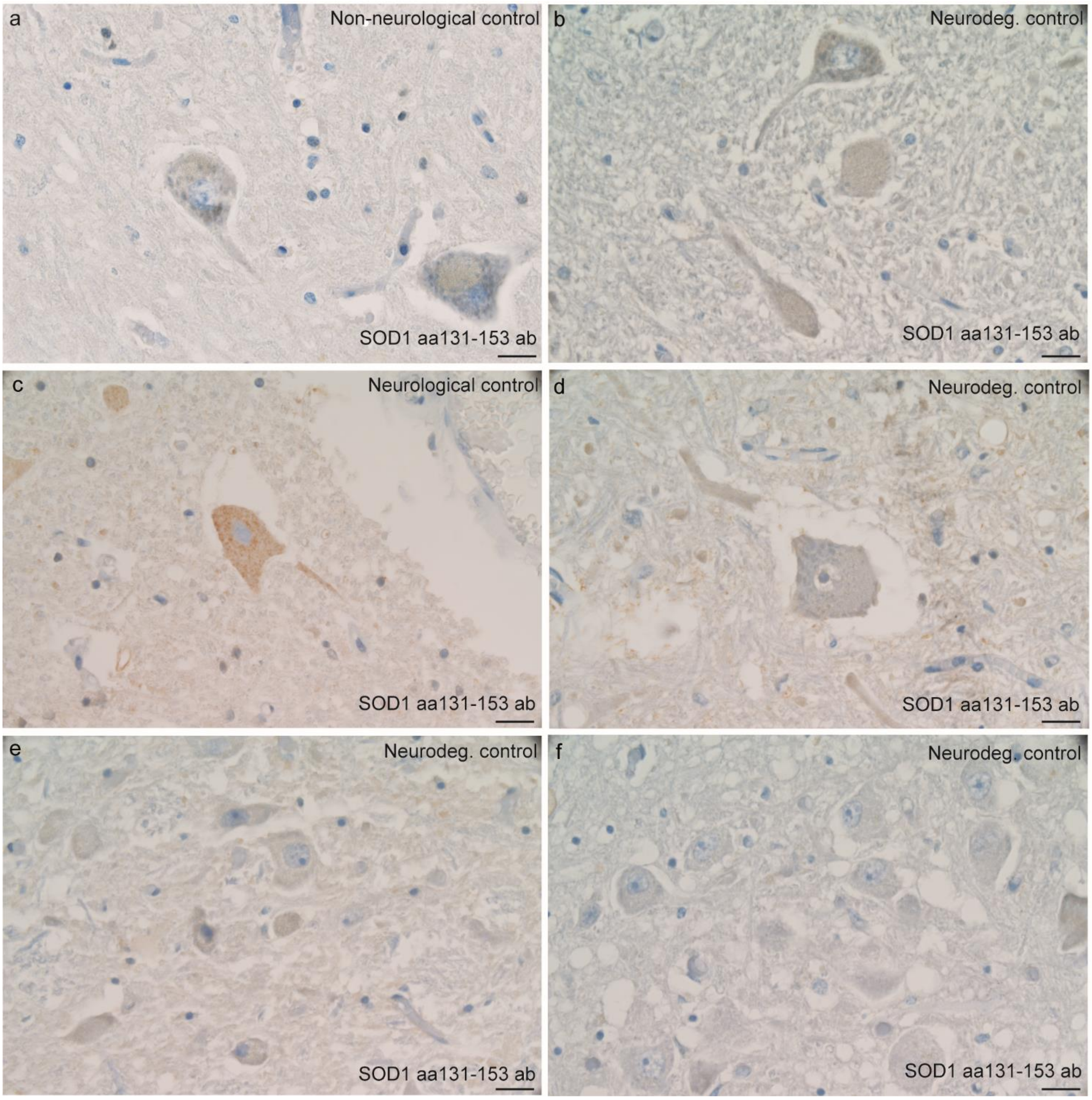

Supplementary Figure 2

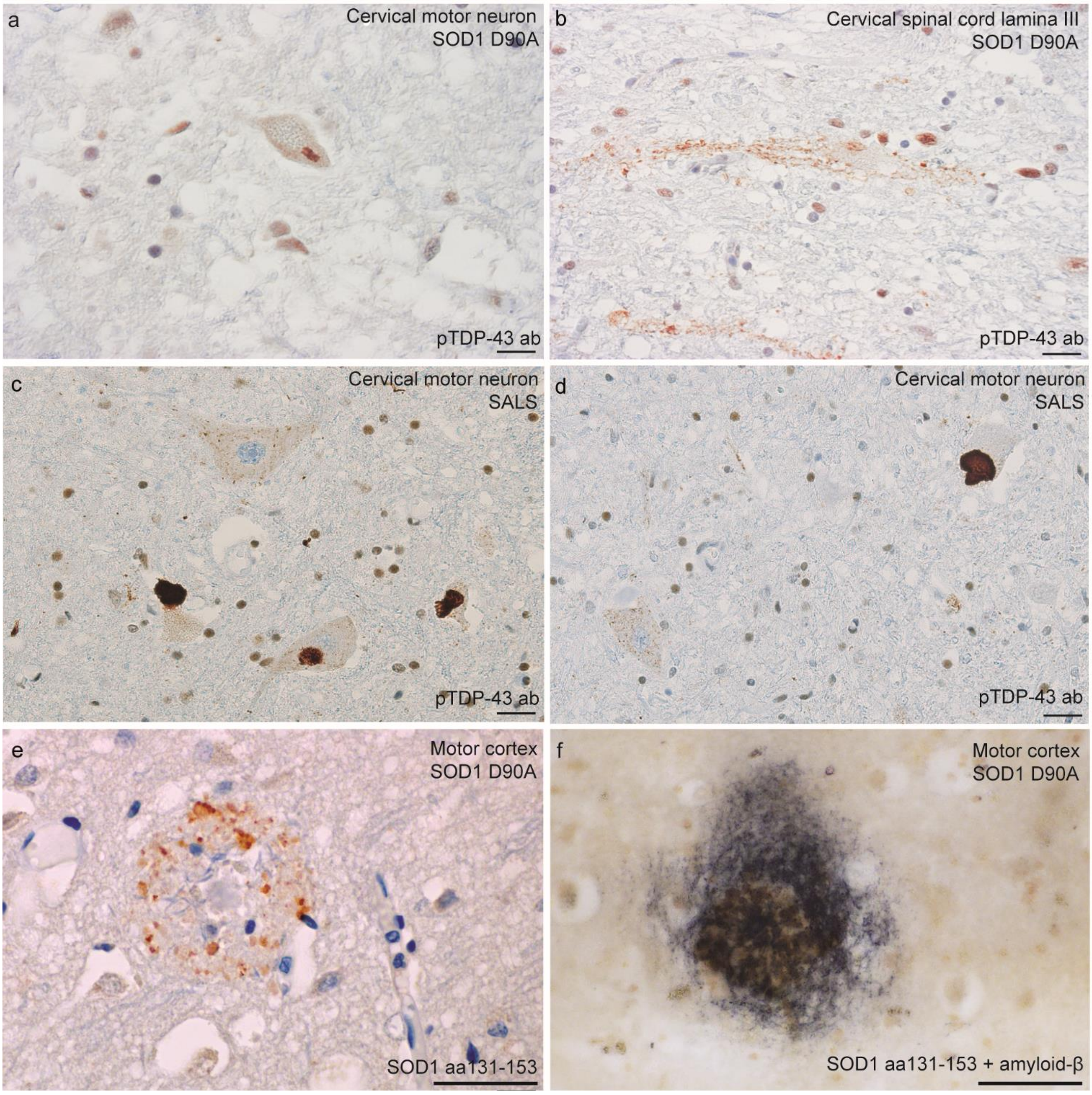

Supplementary Figure 3

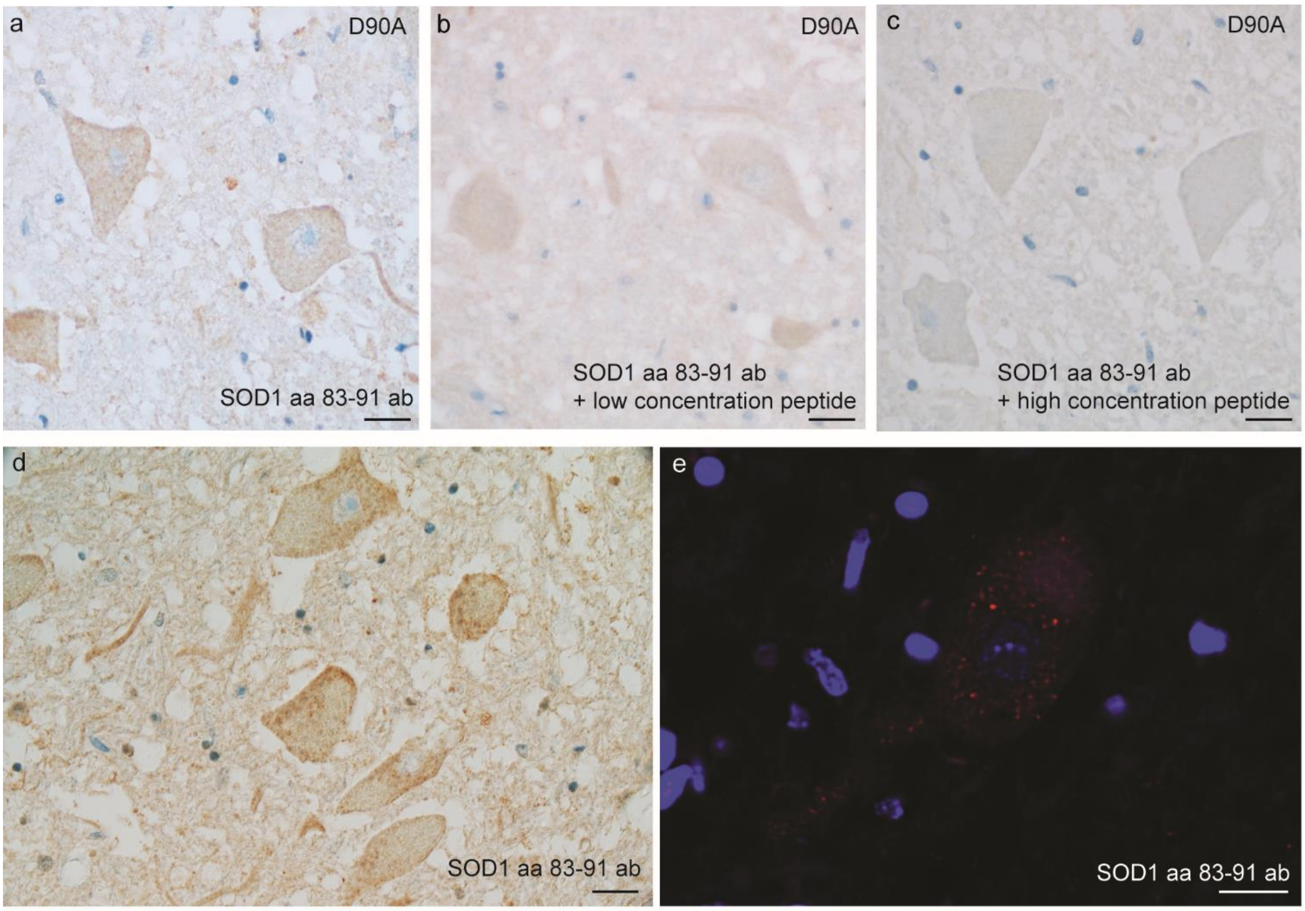

Supplementary Table 1

Case	Cause of death	Age at death	F/M	P.M.	Figures
C #1	Alzheimer´s Disease	54	F	72	Suppl. Fig. 1f
C #2	Parkinson´s Disease	80	F	24	Suppl. Fig. 1b
C #3	Alzheimer´s Disease	77	F	72	
C #4	Status Epilepticus after Stroke	73	M	24	Suppl. Fig. 1c
C #5	Vascular dementia	81	F	48	
C #6	Parkinson´s Disease	83	M	72	
C #7	Myocardial infarction	80	F	N.A	Suppl. Fig. 1a
C #8	Alzheimer´s Disease	86	M	24	
C #9	Parkinson´s Disease	75	M	72	Suppl. Fig. 1d
C #10	Parkinson´s Disease	87	M	48	Suppl. Fig. 1e
ALS					
S #1	Respiratory insufficiency (ALS)	69	F	48	
S #2	Pneumonia (ALS)	82	F	48	
S #3	Respiratory insufficiency (ALS)	62	F	72	Suppl. Fig 2d
S #4	Respiratory insufficiency (ALS)	77	M	72	
S #5	Pneumonia (ALS)	65	F	N.A.	
S #6	Respiratory insufficiency (ALS)	68	M	72	Fig. 1h
S #7	Pneumonia (ALS)	71	M	48	
S #8	Pulmonary embolism (ALS)	63	F	24	Suppl. Fig 2c
F #9	Pneumonia (ALS)	62	M	48	Fig. 1i
F #10	Respiratory insufficiency (ALS)	70	M	48	

Cases C #1-10 are neurodegenerative controls, cases S #1-8 are patients with sporadic ALS and F #9-10 are familial ALS patients with pathogenic *C9orf72HRE* mutation. Age at death (years), F = female, M = Male, P.M., Post mortem time (in hours), N.A., not applicable

Supplementary Table 2

Description	Product identification	Final antibody concentration	Dilution	Manufacturer
<u>Primary antibodies</u>				
Ubiquitin	Z5116	3.8 µg/µl		Dako, Glostrup, Denmark
pTDP-43	22309–1-AP		1:500	Proteintech, Rosemont, IL, USA
p62	610832	5 µg/ml		BD Biosciences, San José, CA, USA
GFAP	Z0334	2.7 µg/ml		Dako, Glostrup, Denmark
Cystatin C	A0451	8 µg/µl		Dako, Glostrup, Denmark
Tau protein (clone AT8)	MN1020	6 µg/ml		Invitrogen, Thermo Fisher
α-synnuclein (clone KM51)	NCL-ASYN		1:20	Novocastra Laboratories
Beta-amyloid	6F/3D, M0872		1:100	Dako, Glostrup, Denmark
Myosin slow	M08421		1:1000	Sigma Aldrich
<u>Secondary antibodies</u>				
Goat anti-rabbit IgG[2]	A-11008	20 µg/ml		Molecular Probes, Eugene, OR, USA
Goat anti-mouse IgG[4]	A-21422	20 µg/ml		Molecular Probes, Eugene, OR, USA

Supplementary Table 3

Grading of pTDP-43 staining in the nine patients with *SOD1*^{D90Ahom} , eight patients with sALS and two fALS patients heterozygous for *C9orf72HRE.*

	Motor system	dorsal horn lamina I-IV	
	LMN	neurons	neuropil
SOD1^{D90A} #1	0	0	0
SOD1^{D90A} #2	0	0	0
SOD1^{D90A} #3	+	0	+
SOD1^{D90A} #4	0	0	0
SOD1^{D90A} #5	0	0	+
SOD1^{D90A} #6	0	0	+
SOD1^{D90A} #7	0	0	0
SOD1^{D90A} #8	0	0	0
SOD1^{D90A} #9	0	0	0
sALS #1	++	+	+
sALS #2	++	0	+
sALS #3	++	+	+
sALS #4	++	+	+
sALS #5	++	0	0
sALS #6	++	0	0
sALS #7	++	0	+
sALS #8	++	0	0
fALS #9	++	+	+
fALS #10	++	0	0
